# A critical response to recent publications on gender affirming care

**DOI:** 10.1177/10398562251393778

**Published:** 2025-11-03

**Authors:** Cláudia C Gonçalves, Marko Milicevic, Alison R Yung

**Affiliations:** Institute for Mental and Physical Health and Clinical Translation, Deakin University, Geelong, VIC, Australia; Institute for Mental and Physical Health and Clinical Translation, Deakin University, Geelong, VIC, Australia; School of Health Sciences, University of Manchester, Manchester, UK

Dear Editor,

A recent issue of *Australasian Psychiatry* (*Vol.* 33, Issue 2, April 2025) included a section titled ‘Gender Medicine’. This section comprised four articles authored by a combination of the same four authors (hence ‘the authors’), all of whom criticise gender-affirming care (GAC). As a College journal, it is expected that a balanced scientific perspective, including lived experience, would be presented on a highly politicized topic. In this correspondence, we highlight the discrepancies between the authors' claims and current literature and argue against treating opinion pieces as equivalent to evidence-based perspectives on this topic.

While the authors refer to a lack of high-quality evidence supporting GAC, they rarely include robust evidence to support their own arguments. Their reference lists include non-peer-reviewed blog posts and opinion articles. When data is cited, they are selectively presented. For example, the Cass Review, which forms the foundation for many of their arguments, is highly criticised for misusing data and forming conclusions based on speculation rather than evidence.^1^

The authors also critique the lack of randomised controlled trials for gender affirmation. The challenges associated with conducting these have been extensively reported^1,2^ and include concerns around adherence and coercion. There are harms associated with withholding GAC, including invalidation of identity leading to future avoidance of healthcare services.^3^ Given these complexities, well-designed observational and qualitative studies must be enough to demonstrate that GAC is not only safe, but essential to the wellbeing of trans people.

Further, the authors discredit the expertise of both clinicians who work in trans healthcare and individuals with lived experience, implying their bias negates objectivity. We find the opinion that all expertise is biased concerning, as high-quality research cannot exist separately from the context in which it is applied, nor the groups whom it affects.

Moreover, the authors ignore evidence indicating positive outcomes related to gender affirmation. This includes reviews demonstrating reductions in suicidal ideation, anxiety, depression, and increased body image satisfaction and quality of life.^4,5^ Further, gender affirmation does not exist in a vacuum, and its benefits are likely dampened by external factors like persistent stigma, a highly politicised climate, and constant negative messaging. External factors are also the leading cause for the small percentage of trans people who ‘detransition’.^6^ If trans people continue to be marginalised and invalidated, both in wider society and within the research landscape, the evidence base for GAC will not demonstrate the full extent of its benefits.

Ultimately, publishing critiques of gender affirmation without offering a balanced view of the literature, including lived experience, does a disservice to the field of psychiatry. We agree that it is imperative to continue researching long-term health outcomes for trans people, including potential negative impacts of hormonal and surgical gender affirmation and ways to mitigate these. However, the continued politicisation of trans identities is obfuscating any potential real harms of existing GAC and leading to challenges in conducting research with these populations. If academic debate allows for the promotion of the view that identifying as trans and seeking gender affirmation is itself a negative outcome, then the goal is no longer about ensuring the wellbeing of trans people. Rather than co-opting this view, research and policy development should be guided by expert voices, including those with lived experience who must live with the consequences of the reality we build.
